# Medial meniscal extrusion: a validation study comparing different methods of assessment

**DOI:** 10.1007/s00167-017-4544-4

**Published:** 2017-04-21

**Authors:** Luke D. Jones, Stephen J. Mellon, Neil Kruger, Andrew P. Monk, Andrew J. Price, David J. Beard

**Affiliations:** Botnar Research Centre, University of Oxford, Windmill Road, Oxford, OX3 7LD UK

**Keywords:** Medial meniscus, Meniscal extrusion, Osteoarthritis, Validation

## Abstract

**Purpose:**

Longitudinal cohort studies of knee OA aetiology use MRI to assess meniscal extrusion within the same knee at sequential time points. A validated method of assessment is required to ensure that extrusion is measured at the same location within the knee at each time point. Absolute perpendicular extrusion from the tibial edge can be assessed using the reference standard of segmentation of the tibia and medial meniscus. This is labour intensive and unsuitable for large cohorts. Two methods are commonly used as proxy measurements. Firstly, the apex of the medial tibial spine is used to identify a reproducible MRI coronal slice, from which extrusion is measured. Secondly, the coronal MRI slice of the knee demonstrating the greatest extrusion is used. The purpose of this study was to validate these two methods against the reference standard and to determine the most appropriate method to use in longitudinal cohort studies. We hypothesised that there is no difference in absolute meniscal extrusion measurements between methods.

**Methods:**

Twenty high-resolution knee MRI scans were obtained in asymptomatic subjects. The tibia and medial meniscus were manually segmented. A custom MATLAB program was used to determine the difference in medial meniscal extrusion of the knee using the reference standard compared to the two other methods.

**Results:**

Assessing extrusion using the single coronal MRI slice demonstrating the greatest extrusion overestimates the true extrusion of the medial meniscus. It incorrectly places the greatest meniscal extrusion at the anterior part of the tibia. Assessing extrusion using a consistent anatomical landmark, such as the medial tibial spine, most reliably corresponds to the reference of segmentation and measurement of true perpendicular extrusion from the tibial edge. Clinicians and researchers should consider this when assessing meniscal extrusion in the knee, and how it changes over time.

**Conclusion:**

This study suggests measuring meniscal extrusion on the coronal MRI slice corresponding to the apex of the medial tibial spine as this correlates most closely with the true perpendicular extrusion measurements obtained from manually segmented models.

**Level of evidence:**

Diagnostic, Level I.

## Introduction

The role of the meniscus in the pathogenesis of painful knee osteoarthritis (OA) is a common target for investigation [1, 7, 22]. As the meniscus “fails”, it extrudes from the joint line and it has been suggested that this is an important step in the development of OA [9]. Large, longitudinal cohort studies of patients, such as the Osteoarthritis Initiative (OAI) [13] and the Multicentre Osteoarthritis Study (MOST) [6], provide opportunities to investigate meniscal extrusion over time by performing MRI scans at sequential time points in the same patient. In these cohorts, MRI scans of the knee are obtained in supine non-weight-bearing position, the same position that scans are obtained in clinical practice. This makes the assessment of these cohorts relevant to the clinical situation. The meniscus is known to behave differently in weight-bearing situations [14, 21]. An absolute extrusion distance of 3 mm or greater than 30% of the total meniscal width is commonly used as the definition of an “extruded” meniscus [8, 11, 15]. An accurate, validated measurement of extrusion is essential to categorise patients appropriately. Segmenting the tibia and meniscus to allow three-dimensional analysis of meniscus has been identified as the most accurate method of determining meniscal extrusion [2, 23, 24] and is considered the reference standard although it is not a suitable technique in large cohorts due to the laborious nature of manual segmentation. Contemporary measurements of meniscal extrusion therefore tend to utilise one of two methods. The first (“Bony Landmarks”) method uses a fixed bony point in the knee on coronal MRI slices, usually the apex of the medial tibial spine [5, 6, 16, 17]. This Bony Landmarks is chosen as it allows the same coronal slice to be reproducibly selected when comparing scans taken on the same patient at different time points. Once the apex of the medial tibial spine has been identified, the horizontal distance between the most medial aspect of the tibia and the most medial aspect of the meniscus on this image is measured. The second (“Coronal Slices”) method inspects all coronal MRI slices and then measures the horizontal distance between the most medial aspect of the tibia and the most medial aspect of the meniscus [7, 8, 11] on the slice that demonstrates the greatest extrusion. Whilst both these methods may be practical, to date neither has been validated as an accurate method of measuring extrusion.

**Fig. 1:**
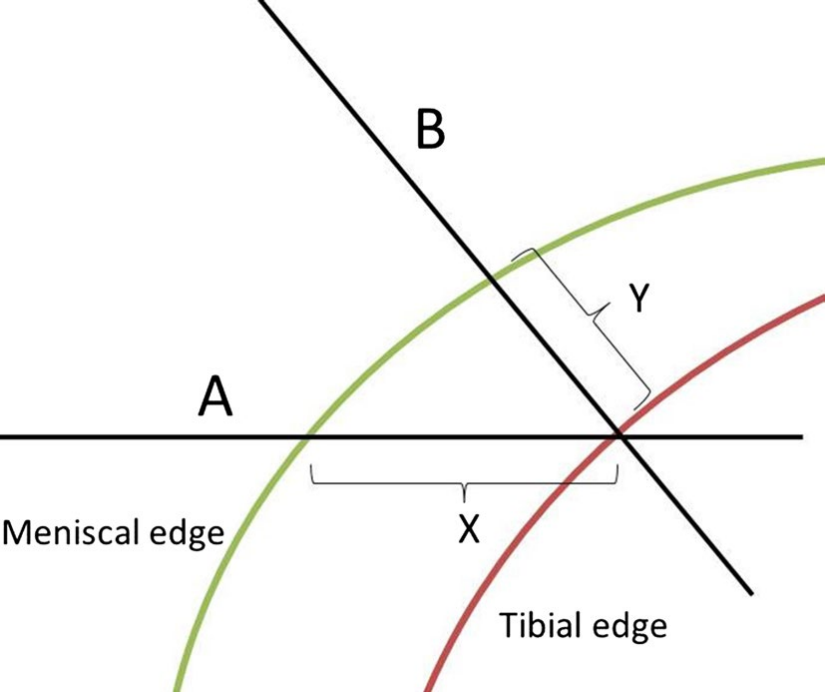
Meniscal extrusion distance assessed on MRI varies depending on whether measurements are taken on a coronal slice or perpendicular to the tibial edge on segmented images

Problems exist with both methods of assessment because both assess the meniscus on one anatomical slice. The “Bony Landmarks” method may lead to false-negative results as menisci that are not extruded at that point but are extruded further anteriorly or posteriorly might be incorrectly labelled as “not extruded”. Similarly, with the “Coronal Slices” method, measuring horizontal extrusion on Coronal Slices anterior and posterior to the midline may falsely increase the perceived “extrusion” as the approximately circular edges of the tibia and meniscus may not be parallel (Fig. 1, line A, distance X).

A more appropriate absolute measurement of extrusion distance would be to assess the perpendicular distance between the edge of the tibia and the edge of the meniscus (Fig. 1, line B, distance Y), although this measurement is only possible following segmentation of the meniscus and tibia—not on Coronal Slices.

In order to determine the most accurate method of measuring medial meniscal extrusion for use in both longitudinal cohorts and clinical assessments, this study aimed to compare the “Bony Landmarks” and “coronal slice” methods to a reference standard—the maximal perpendicular extrusion of the meniscus from the tibial edge calculated from 3D volumetric reconstructions of segmented tibia and meniscus. It was hypothesised that there is no difference in the location of maximal meniscal extrusion or the extent of maximal extrusion when assessed using different measurement techniques compared to the reference standard.

## Materials and methods

Ethical approval was obtained from National Research and Ethics Committee South Central Oxford A (OxRECA 12/SC/006).

High-resolution, supine, 3T MRI scans (Achieva 3.0T X series, Philips Medical Systems International, B.V, Eindhoven, Netherlands) were obtained on 20 asymptomatic patients (20 knees, all males, mean age 28.3 years [SD 6.2]) as part of a larger study into the aetiology of knee OA. The knee was positioned as per a standardised, routine clinical protocol with the patient supine and the ankles supported on foam pads allowing the knee to fall into full extension. With this protocol, the apex of the patella is placed in the centre of the coil, and the knee is held in position with foam pads. A three-plane localiser scan is performed to ensure the correct position of the knee. In the coronal plane, the slices are aligned with the back of the femoral condyles with the joint in the middle of the field of view. The scan then proceeds posterior to anterior from the back of the femoral condyles to the centre of the patella anteriorly. All scans were then reviewed by a single trained observer (LJ) to confirm the absence of osteophytes, degenerative meniscal tears, chondral cartilage lesions and ligamentous insufficiency. In three scans, degenerative cartilage lesions were observed in the medial compartment and these scans were therefore discarded. In total, 17 asymptomatic knees with no degenerative cartilage change in the knee were included in the study. This number of scans is comparable to other studies that have explored segmentation of the meniscus [2]. An a priori power analysis was performed using a power of 0.8, a *p* value of 0.05 and an effect size of 0.5. This indicated a sample size of 11 to be compared across the three methods.

The geometry of each patient’s tibial plateau and meniscus was segmented using Mimics (v. 14.1, Materialise, Belgium) and exported as stereolithography (STL) files. Meniscal segmentation was performed via a method previously validated by Bowers et al. [4], using meniscal volumes calculated by surface integration. Coronal Slices provided near perpendicular views for precise meniscal edge definition at the meniscosynovial rim. Accurate outlining was further aided by the high contrast between the low intrameniscal and high extrameniscal signal intensity on T2-weighted images. 3D meniscal reconstructions were then obtained.

These files were imported into MATLAB (R2010b, The MathWorks Inc., Natick, MA, USA), and medial meniscal extrusion was calculated using the following technique. Firstly, the tibia and meniscus geometry was rotated to give a view down the longitudinal axis of the tibia. The breadth of the tibial plateau and the highest point (the apex of the medial tibial spine) were then determined.

**Fig. 2:**
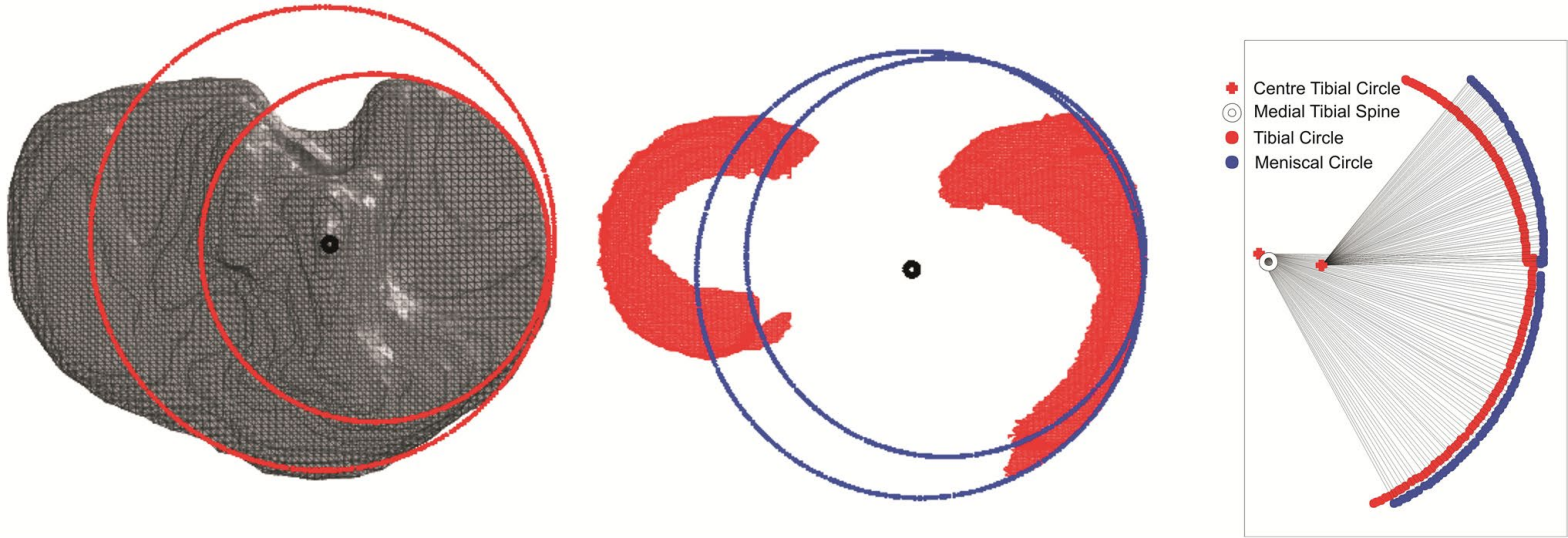
CETCEM method of assessing meniscal extrusion

A “Circular Edge of Tibia to Circular Edge of Meniscus” (CETCEM) algorithm was then used to establish the reference standard method. A coronal plane through the medial tibial spine was used to divide the tibia and meniscus into anterior and posterior portions creating four distinct anatomical regions. Multiple points on the medial edge of the anterior and posterior tibia and anterior and posterior meniscus were selected, and circles were fitted to these points. The edges of both the meniscus and tibia were linearly interpolated to fill gaps caused by the scan slice thickness. Vectors were projected from the centre of the anterior and posterior tibial circles to corresponding points on the circumference of the circles fitted to the anterior and posterior portions of the meniscus (Fig. 2). Extrusion was calculated by subtracting the radius of the tibial circles from the length of the vectors. As this represented the true perpendicular extrusion of the meniscus from the tibial edge, this was used as the reference standard with which to compare the other methods of assessment.

**Fig. 3:**
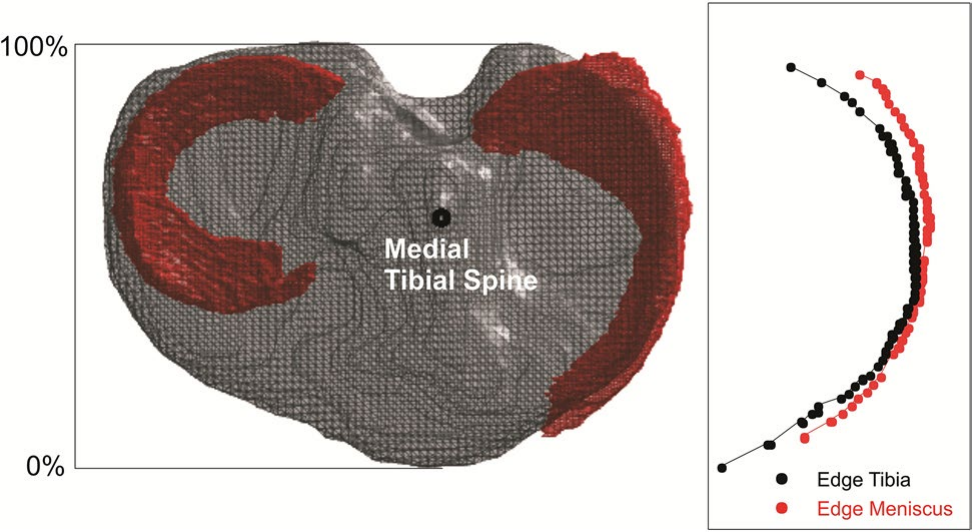
Coronal Slices method of assessing meniscal extrusion using horizontal distance between the most medial edge of the meniscus and the most medial edge of the tibia

For the comparator groups (“Coronal Slices” and “Bony Landmarks”), measurements were carried out on slices at 1-millimetre intervals from the front to the back of the tibial plateau in the AP plane (simulating Coronal Slices on an MRI scan). Extrusion was calculated as the horizontal difference between the most medial edge of the meniscus and the tibia (Fig. 3) at each 1-mm interval. Measurements of meniscal extrusion were made on the simulated slice containing the apex of the medial tibial spine (representing the “Bony Landmarks” method), as well as the simulated slice with the maximal horizontal extrusion measured when comparing each 1-mm AP interval (representing the “Coronal Slices” method).

To allow comparison of extrusion between patients (and therefore between tibias of different sizes), the position of the measurements was expressed as a percentage of the AP depth of the tibial plateau, i.e. 0% corresponded to the front (most anterior point of joint line) of the tibia, as well as in absolute distance in millimetres from the front of the tibia.

### Statistical analysis

Five knee scans were read on two occasions by two observers to allow assessment of intra- and interobserver reliability, and statistical analysis was performed using SPSS v18.0.0 (IBM, NY, USA). A Wilcoxon signed rank test and Friedman’s ANOVA for nonparametric data were used to assess the location and maximal meniscal extrusion, and a bivariate correlation analysis was used to determine agreement between methods.

## Results

Using the commonly accepted definition of extrusion as 3 mm, and then defining the meniscus as either extruded or not, intraobserver reliability studies for the binary extrusion of the meniscus using all three methods demonstrated a kappa value of 1.0 (SE 0.00, *p* = 0.00) indicating perfect agreement. Interobserver reliability studies for the binary extrusion of the meniscus using all three methods demonstrated a kappa value of 0.82 (SE 0.16 *p* = 0.00) indicating substantial agreement.

The mean AP tibial breadth was 52.9 mm (range 47.2–61.6 mm, SD 3.6), and the highest point of the medial tibial spine was found at mean 29.8 mm (range 28–33 mm, SD 1.7) from the most anterior point of the tibial plateau. This corresponded to 56% of the AP tibial breadth and was the anatomical location of the slice used in the “Bony Landmarks” method.

Using the “Coronal Slices” method, the median maximal location of horizontal extrusion was found to be 10.8 mm (21%, range 2.8–53.1 mm, SD 18.2, mean 20.3 mm) from the front of the tibial plateau. Using the CETCEM reference method, the median maximal location of horizontal extrusion was found to be 26.0 mm (48%, range 3–53 mm, SD 15.4, mean 26.7 mm) from the front of the tibial plateau. A Wilcoxon signed rank test for non-normally distributed data revealed a significant difference (*p* = 0.02) between the measurement methods. The location of maximal meniscal extrusion was consistently measured as more anterior by the “Coronal Slices” method and more posterior by the “Bony Landmarks” method when compared to the reference CETCEM method.

The median maximum meniscal extrusion was 4.8 mm (SD 1.7, mean 4.7) when measured by the “Coronal Slices” method, compared to 3.1 mm (SD 1.2, mean 3.0) when measured by the CETCEM method. The median maximum meniscal extrusion measured using the “Bony Landmarks” method was 2.2 mm (SD 1.5, mean 2.4).

A Friedman’s ANOVA for nonparametric data indicated that the maximum meniscal extrusion was significantly higher when measured using the “Coronal Slices” method compared to both the CETCEM method (4.8 vs 3.1 mm) and the “Bony Landmarks” method (4.8 vs 2.2 mm) (*p* < 0.001). In addition, there was a statistically significant difference between the estimated maximum extrusion using the CETCEM method as compared with the “Bony Landmarks” method (3.1 vs 2.2 mm) (*p* < 0.001).

Bivariate correlation analysis of the maximum extrusion between measurement techniques indicates Spearman’s rho correlation coefficients between the CETCEM method and “Coronal Slices” method of *r* = 0.64 (*p* = 0.04), and between the “Bony Landmarks” and CETCEM method of *r* = 0.72 (*p* < 0.001). The “Bony Landmarks” and the “Coronal Slices” method corresponded poorly, *r* = 0.53 (*p* = 0.03).

## Discussion

The most important finding of this validation study comparing different methods of measuring meniscal extrusion is that maximum medial meniscal extrusion does not occur reliably in one location along the medial edge of the tibia (in the AP plane), irrespective of the measurement method used. The “Coronal Slices” method, as expected, found the location of maximal extrusion to be very anterior on the tibia compared to the reference CETCEM method and the “Bony Landmarks” method. In addition, the “Coronal Slices” method overestimated maximal extrusion compared to the reference CETCEM method. The “Bony Landmarks” method tended to underestimate maximal extrusion. However, the maximal extrusion measured using the “Bony Landmarks” method correlated more closely with the reference CETCEM method than the maximal extrusion measured using the “Coronal Slices” method.

The role of the pathological and normal meniscus in the pathogenesis of osteoarthritis of the knee has been debated for some time. The link between joint space narrowing and meniscal extrusion in symptomatic knee OA was first explored by Gale et al. [7] who demonstrated that meniscal subluxation is strongly associated with symptomatic knee OA, suggesting that joint space narrowing was secondary to extrusion and not hyaline cartilage loss. They postulated that, upon extrusion, the meniscus fails to perform its primary function of distributing loads across the hyaline cartilage leading to increased contact pressures and subsequent cartilage degeneration. Thus, meniscal extrusion has become a focus for clinical and epidemiological investigators. The importance of a validated method of assessment, particularly in longitudinal studies that inspect the same knee at different time points, is clear. The use of meniscal extrusion measurements in the assessment of the efficacy of both meniscal transplant and meniscal repair techniques is also common place [3, 10, 12, 18–20, 25].

This is the first time to our knowledge that different methods of measurement of extrusion have been directly compared in the same knee. This study indicates that if investigators choose to assess meniscal extrusion using “Coronal Slices” method, they will consistently overestimate the maximum extrusion distance. This occurs because the edge of the tibia and the meniscus is not parallel anterior and posterior to the midline, and therefore, measurements are not made perpendicular to the tibial edge (Fig. 1). It also suggests that, as the CETCEM reference method demonstrates, maximal extrusion occurs at a point 26.0 mm (48%) from the front of the knee. Measuring meniscal extrusion at the level of the highest point of the medial tibial spine is therefore a reasonable proxy for the more labour-intensive CETCEM method.

There are limitations to this study. The MRI scans utilised in this study were taken in a supine position, and it is well established that the meniscus behaves differently under weight-bearing conditions [21]. However, the aim of this study was to determine the most suitable method for assessing meniscal extrusion for use in both longitudinal cohorts and in assessing outcome of interventions such as meniscal transplantation. In clinical practice, MRI scans are obtained in the supine non-weight-bearing position. Similarly, MRI scans are obtained supine in large longitudinal cohorts such as the Osteoarthritis Initiative. Hence, this study is applicable for both these uses. Further studies on the location of meniscal extrusion on the weight-bearing scan should be performed when this method of imaging is used more commonly in the clinical setting. The results of this study should not be extrapolated weight-bearing scenario. This study has only considered those with no degenerative change in the knee. It may be that the meniscus in a degenerative knee does not behave in the same way [12]. For example, the presence of medial tibial osteophyte may act as a physical barrier to limit extrusion.

The CETCEM method requires the tibia and the meniscus to be segmented. This makes it unsuitable for longitudinal datasets as manual segmentation is time-consuming and requires specialist software. In this study, the CETCEM method was used as a theoretical “reference standard” against which other methods can be compared. This study indicates that the “Coronal Slices” method tends to overestimate extrusion and should not be used as an assessment method. Clinicians assessing longitudinal change in meniscal extrusion, whether as an indicator of impending OA or to determine the long-term success of meniscal transplantation, should be aware of the limitations of the “Coronal Slices” method and assess extrusion at the level of the medial tibial spine.

## Conclusion

In cross-sectional MRI imaging of the medial meniscus of the knee, assessing extrusion at the level of the apex of the medial tibial spine represents a pragmatic and validated alternative to the reference standard method of segmentation.

## References

[1] Bae JY, Park KS, Seon JK, Kwak DS, Jeon I, Song EK (2012). Biomechanical analysis of the effects of medial meniscectomy on degenerative osteoarthritis. Med Biol Eng Comput.

[2] Bloecker K, Guermazi A, Wirth W, Benichou O, Kwoh CK, Hunter DJ, Englund M, Resch H, Eckstein F (2013). Tibial coverage, meniscus position, size and damage in knees discordant for joint space narrowing. Data from the Osteoarthritis Initiative. Osteoarthr Cartil.

[3] Bloecker K, Wirth W, Guermazi A, Hunter D, Resch H, Hochreiter J, Eckstein F (2015). Medial meniscal extrusion relates to cartilage loss in specific femorotibial subregions—data from the Osteoarthritis Initiative. Arthritis Care Res.

[4] Bowers ME, Tung GA, Fleming BC, Crisco JJ, Rey J (2007). Quantification of meniscal volume by segmentation of 3T magnetic resonance images. J Biomech.

[5] Crema MD, Guermazi A, Li L, Nogueira-Barbosa MH, Marra MD, Roemer FW, Eckstein F, Le Graverand MP, Wyman BT, Hunter DJ, Cremayzxk MD, Guermaziyz A, Li L, Marrayz MD, Roemeryz FW, Ecksteinyy F, Le Graverandzz MPH, Wymanzz BT, Hunter DJ (2010). The association of prevalent medial meniscal pathology with cartilage loss in the medial tibiofemoral compartment over a 2-year period. Osteoarthr Cartil.

[6] Englund M, Guermazi A, Roemer FW, Yang M, Zhang Y, Nevitt MC, Lynch JA, Lewis CE, Torner J, Felson DT (2010). Meniscal pathology on MRI increases the risk for both incident and enlarging subchondral bone marrow lesions of the knee: the MOST Study. Ann Rheum Dis.

[7] Gale DR, Chaisson CE, Totterman SMS, Schwartz RK, Gale ME, Felson D (1999). Meniscal subluxation: association with osteoarthritis and joint space narrowing. Osteoarthr Cartil.

[8] Ha JJK, Shim JCJ, Kim DWD, Lee YSY, Ra HHJ, Kim JJG (2010). Relationship between meniscal extrusion and various clinical findings after meniscus allograft transplantation. Am J Sports Med.

[9] Hunter D (2012). Degeneration of the meniscus and progression of osteoarthritis. Musculoskelet J Hosp Spec Surg.

[10] Lee B-S, Bin S-I, Kim J-M, Kim JH, Lim EJ (2017). Meniscal allograft subluxations are not associated with preoperative native meniscal subluxations. Knee Surg Sports Traumatol Arthrosc.

[11] Lee D, Kim S, Kim T, Cha E, Bin S (2010). Midterm outcomes after meniscal allograft transplantation. Am J Sports Med.

[12] Lee DH, Lee BS, Kim JM, Yang KS, Cha EJ, Park JH, Il Bin S (2011). Predictors of degenerative medial meniscus extrusion: radial component and knee osteoarthritis. Knee Surg Sports Traumatol Arthrosc.

[13] Lester G (2012). The Osteoarthritis Initiative: a NIH public–private partnership. HSS J.

[14] Paparo F, Revelli M, Piccazzo R, Astengo D, Camellino D, Puntoni M, Muda A, Rollandi GA, Garlaschi G, Cimmino MA (2015). Extrusion of the medial meniscus in knee osteoarthritis assessed with a rotating clino-orthostatic permanent-magnet MRI scanner. Radiol Med.

[15] Rennie WWJ, Finlay DBL (2006). Meniscal extrusion in young athletes: associated knee joint abnormalities. Am J Roentgenol.

[16] Roemer FW, Felson DT, Nevitt MC, Marra MD, Torner JC, Lewis CE, Guermazi A, Crema MD, Roemer FW, Felson DT, Englund M, Wang K, Jarraya M, Nevitt MC, Marra MD, Torner JC, Lewis CE, Guermazi A (2012). Factors associated with meniscal extrusion in knees with or at risk for osteoarthritis: the Multicenter Osteoarthritis study. Radiology.

[17] Roemer FW, Lynch JA, Crema MD, Marra MD, Nevitt MC, Felson DT, Hughes LB, El-khoury GY (2009). Tibiofemoral joint osteoarthritis: risk factors for MR-depicted fast cartilage loss over a 30-month period in the Multicenter Osteoarthritis study. Radiology.

[18] Roubille C, Martel-Pelletier J, Abram F, Dorais M, Delorme P, Raynauld JP, Pelletier JP (2015). Impact of disease treatments on the progression of knee osteoarthritis structural changes related to meniscal extrusion: data from the OAI progression cohort. Semin Arthritis Rheum.

[19] Schuttler K, Haberhauer F, Gesslein M, Heyse T, Figiel J, Lorbach O, Efe T, Roessler P (2016). Midterm follow-up after implantation of a polyurethane meniscal scaffold for segmental medial meniscus loss: maintenance of good clinical and MRI outcome. Knee Surg Sports Traumatol Arthrosc.

[20] Schuttler K, Pottgen S, Getgood A, Rominger M, Fuchs-Winkelmann S, Roessler P, Ziring E, Efe T (2015). Improvement in outcomes after implantation of a novel polyurethane meniscal scaffold for the treatment of medial meniscus deficiency. Knee Surg Sports Traumatol Arthrosc.

[21] Stehling C, Souza RB, Le Graverand MPH, Wyman BT, Li X, Majumdar S, Link TM (2012). Loading of the knee during 3.0 T MRI is associated with significantly increased medial meniscus extrusion in mild and moderate osteoarthritis. Eur J Radiol.

[22] Sugita T, Kawamata T, Ohnuma M, Yoshizumi Y, Sato K (2001). Radial displacement of the medial meniscus in varus osteoarthritis of the knee. Clin Orthop Relat Res.

[23] Wenger A, Englund M, Wirth W, Hudelmaier M, Kwoh K, Eckstein F (2012). Relationship of 3D meniscal morphology and position with knee pain in subjects with knee osteoarthritis: a pilot study. Eur Radiol.

[24] Wenger A, Wirth W, Hudelmaier M, Noebauer-Huhmann I, Trattnig S, Bloecker K, Frobell RB, Kwoh K, Eckstein F, Englund M (2013). Meniscus body position, size and shape in persons with and without radiographic knee osteoarthritis: quantitative analyses of knee MRIs from the Osteoarthritis Initiative. Arthritis Rheum.

[25] Zhang F, Kumm J, Svensson F, Turkiewicz A, Frobell R, Englund M (2016). Risk factors for meniscal body extrusion on MRI in subjects free of radiographic knee osteoarthritis: longitudinal data from the Osteoarthritis Initiative. Osteoarthr Cartil.

